# ATR-FTIR Microspectroscopy Brings a Novel Insight Into the Study of Cell Wall Chemistry at the Cellular Level

**DOI:** 10.3389/fpls.2020.00105

**Published:** 2020-02-21

**Authors:** Clément Cuello, Paul Marchand, Françoise Laurans, Camille Grand-Perret, Véronique Lainé-Prade, Gilles Pilate, Annabelle Déjardin

**Affiliations:** ^1^ INRAE, ONF, BioForA, Orléans, France; ^2^ INRAE, Phenobois, Orléans, France

**Keywords:** ATR-FTIR microspectroscopy, phenotyping, poplar, cell wall, G-layer, wood

## Abstract

Wood is a complex tissue that fulfills three major functions in trees: water conduction, mechanical support and nutrient storage. In Angiosperm trees, vessels, fibers and parenchyma rays are respectively assigned to these functions. Cell wall composition and structure strongly varies according to cell type, developmental stages and environmental conditions. This complexity can therefore hinder the study of the molecular mechanisms of wood formation, underlying the construction of its properties. However, this can be circumvented thanks to the development of cell-specific approaches and microphenotyping. Here, we present a non-destructive microphenotyping method based on attenuated total reflectance–Fourier transformed infrared (ATR-FTIR) microspectroscopy. We applied this technique to three types of poplar wood: normal wood of staked trees (NW), tension and opposite wood of artificially tilted trees (TW, OW). TW is produced by angiosperm trees in response to mechanical strains and is characterized by the presence of G fibers, exhibiting a thick gelatinous extra-layer, named G-layer, located in place of the usual S2 and/or S3 layers. By contrast, OW located on the opposite side of the trunk is totally deprived of fibers with G-layers. We developed a workflow for hyperspectral image analysis with both automatic pixel clustering according to cell wall types and identification of differentially absorbed wavenumbers (DAWNs). As pixel clustering failed to assign pixels to ray S-layers with sufficient efficiency, the IR profiling and identification of DAWNs were restricted to fiber and vessel cell walls. As reported elsewhere, this workflow identified cellulose as the main component of the G-layers, while the amount in acetylated xylans and lignins were shown to be reduced. These results validate ATR-FTIR technique for *in situ* characterization of G layers. In addition, this study brought new information about IR profiling of S-layers in TW, OW and NW. While OW and NW exhibited similar profiles, TW fibers S-layers combined characteristics of TW G-layers and of regular fiber S-layers. Unexpectedly, vessel S-layers of the three kinds of wood showed significant differences in IR profiling. In conclusion, ATR-FTIR microspectroscopy offers new possibilities for studying cell wall composition at the cell level.

## Introduction

Trees are able to live long and reach a considerable height, partly thanks to the remarkable properties of their wood. Indeed, wood—or secondary xylem—fulfills three major functions in trees: (i) water conduction from the roots to the crown, (ii) mechanical support of the ever-increasing mass of the growing tree submitted to different environmental cues (wind, slope, light, …), and (iii) storage of temporary reserves, important for tree perennial growth (Plomion et al., 2001; Déjardin et al., 2010). In Angiosperm trees, vessels, fibers and parenchyma rays are respectively assigned to each of these functions. These different xylem cell types originate from the differentiation of cambial cells. When reaching their final dimensions, xylem cells build a secondary cell wall that will be deposited onto the primary wall and middle lamella. Vessels are rapidly submitted to programmed cell death after lignification of their cell walls, then fibers have the same destiny (Courtois‐Moreau et al., 2009), while ray cells remain alive for several years (Nakaba et al., 2012). Therefore, wood results in the complex assembly of the cell walls of dead fibers and vessels, connected to living parenchyma rays (Déjardin et al., 2010).

Wood cell walls exhibit a multi-layered structure, determinant for wood mechanical properties: middle lamella and primary cell wall are overlaid by three layers of secondary cell walls (SCW), named S1, S2, and S3, S2 being the thickest. Each layer results from the assembly of semi-crystalline cellulose microfibrils embedded in an amorphous matrix of polysaccharides and lignins. They differ according to cellulose microfibril orientation and the nature and proportion of the matrix components (pectins, hemicelluloses and lignins) (Mellerowicz and Sundberg, 2008). The middle lamella and primary cell wall are known to be rich in lignins, pectins and xyloglucans, with apparently cellulose microfibrils oriented at random. By contrast, the S-layers are rich in xylans with oriented cellulose microfibrils: the angle formed between the cell axis and cellulose microfibrils, named microfibril angle (MFA) is wide in the S1- and S3-layers, while it is fairly low in the S2-layer (Barnett and Bonham, 2004). At the end of cell wall deposition, lignification occurs, leading to the impermeabilization of the cell walls. Lignins are phenolic polymers merely resulting, in hardwood species, from the polymerization of two classes of monolignols (coniferyl alcohol or G unit and synapyl alcohol or S unit) that differ in their degree of methoxylation (Freudenberg and Neish, 1968). They are synthesized in the cytoplasm, then transported to the cell wall, turned into chemical radicals by the activity of laccases and peroxidases and probably get polymerized in a random manner. Lignin structure is therefore highly diverse and difficult to predict (van Parijs et al., 2010).

Wood cell walls are highly variable according to developmental processes or various environmental stresses. First, it is known that secondary cell wall composition differs between vessels, fibers and ray cells (e.g. Donaldson et al., 2001). The cell wall of vessel elements is enriched in G-units while fiber cell wall is richer in S-units (Terashima et al., 1993). Wood cell wall composition can also be affected by various environmental cues. For example, in response to mechanical strains, Angiosperm trees produce tension wood on the upper side of inclined trunks or branches in order to reorient them or at least to reach a new equilibrium. In poplar, tension wood fibers exhibit a thick gelatinous extra-layer, named G-layer (Jourez, 1997). Located in place of the usual S2 and/or S3 layers, G-layer is rich in cellulose, rhamnogalacturonan type I pectins (RG-I), and arabinogalactan proteins (AGP) (Gorshkova et al., 2015; Guedes et al., 2017), with nearly no lignin (Joseleau et al., 2004; Pilate et al., 2004; Gierlinger and Schwanninger, 2006). In G-layer, cellulose is more crystalline and MFA is close to 0° (Norberg and Meier, 1966; Gorshkova et al., 2015).

Thus, wood is both a highly complex and variable tissue, which does not facilitate the study of the molecular mechanisms underlying its formation and the building of its properties. However, this hindrance may be alleviated thanks to the development of cell-specific approaches and microphenotyping. Particularly, the development of microphenotyping methods may provide extensive biochemical information at the cell wall level. In the last decade, Fourier Transform InfraRed (FTIR) spectroscopy made possible high throughput biochemical analyses of lignocellulosic biomass. Indeed, the wavenumbers emitted by infrared beams are differentially absorbed according to the chemical bonds, therefore the functional groups, present in the sample. Cell wall composition may be determined by prediction equations based on near-infrared absorbance spectra of ground plant materials. This technique has been successfully used to predict cell wall composition in *Arabidopsis thaliana* (Jasinski et al., 2016), forage crops (Fairbrother and Brink, 1990; Molano et al., 2016; Baldy et al., 2017; Li et al., 2017), rice (Huang et al., 2017) and poplar (Gebreselassie et al., 2017). Mid-infrared (MIR) spectroscopy is complementary to predictive near-infrared (NIR) spectroscopy. Indeed, MIR spectroscopy targets the fundamental vibrations of the molecules, which gives a good visualization of the molecular bonds present in the sample (Bertrand and Dufour, 2006; Allison, 2011) and also to assign variations in the absorbance at a given wavenumber to a class of compounds (McCann et al., 1992; Kacurakova and Wilson, 2001). For example, FTIR spectroscopy was used to discriminate between different Arabidopsis cell wall mutants (Chen et al., 1998). FTIR spectroscopy coupled with microscopy imaging makes possible *in situ* analysis of cell wall assembly (Mouille et al., 2003; Gierlinger et al., 2008; Gorzsás et al., 2011; Chazal et al., 2014; Gierlinger, 2018).

In this methodological study, we present a non-destructive high-throughput microphenotyping method based on ATR-FTIR microspectroscopy, one of the most resolving methods currently available. The objective was to develop a method to obtain IR spectra specific to wood cell types, fibers, vessels and rays. Therefore, we have developed a high-throughput pipeline for hyperspectral image analysis with both automatic pixel clustering and identification of differentially absorbed wavenumbers between samples. We have implemented this pipeline on different types of wood, including tension wood, a model that has been extensively characterized from a biochemical point of view. We confirmed previously known results, which validates this *in situ* approach, and we generated for the first time an IR profile for vessel walls in three different types of wood.

## Materials and Methods

### Plant Material and Sample Preparation

The study was carried out on six three-month-old ramets, originating from *in vitro* micropropagated shoots of the INRA 717-1B4 clone (*Populus tremula* L. x *Populus alba* L.). Two ramets were grown in a greenhouse in spring 2011 and the four others in the autumn and winter 2012. In the latter case, the plants were supplemented with sufficient amount of artificial light to keep the cambium active. In order to induce the production of TW, three of these trees (TT_11-A in 2011, TT_12-A and TT_12-B in 2012) were tilted at 45° two months before sampling. The three other poplar plants (UT_11-C, UT_12-C and UT_12-D) were grown upright in the same conditions and produce no or very few TW. Three-centimeter-long stem fragments were sampled at the base of each tree. NW was collected on upright trees, TW and OW on the upper and lower side of artificially tilted trees, respectively. The samples were stored at –20°C until use.

For each tree, 20 µm-thick cross-sections were cut from the frozen wood samples using a RM2155 microtome (LEICA, Wetzlar, Germany). The sections were ethanol-dried between two glass slides to ensure flatness and stored at 23°C and 45% RH until ATR-FTIR analysis.

### Hyperspectral Imaging

ATR-FTIR images were produced from stem cross-sections by a mid-IR light (1,800–850 cm^–1^) at a 4 cm^–1^ spectral resolution using a Spotlight 400 FTIR imaging system coupled to a Spectrum 400 FTIR spectrophotometer (PERKIN ELMER, Wellesley, USA). According to the manufacturer’s procedure, the wood section was pushed by pressure into direct contact with the tip of the 600-µm diameter plane Germanium crystal. **Supplementary Figure 1** shows the contact area after pressure release showing the intimate contact of the sample with the crystal. Sixteen scans per pixel were taken in order to enhance signal-to-noise ratio. For each type of wood (TW, OW and NW), three (100 x 100) µm² images from each cross section were taken at a (1.56 x 1.56) µm² pixel size. The acquisition time of an image of this size was 40 minutes. This maximal pixel resolution corresponded to an oversampling factor of two, compared with the diffraction-limited spatial resolution of 3.1 µm. At this pixel resolution, the image size was 4,096 pixels. Therefore, for each type of wood, the data set was composed of 36,864 spectra (3 different trees x 3 images x 4,096 pixels). The spectra were then corrected using different functions of the SpectrumImage software (v.1.6.4): background correction during acquisition, noise reduction and atmospheric correction, using default parameters. No ATR correction was performed.

### Multivariate Image Analysis

**Figure 1:**
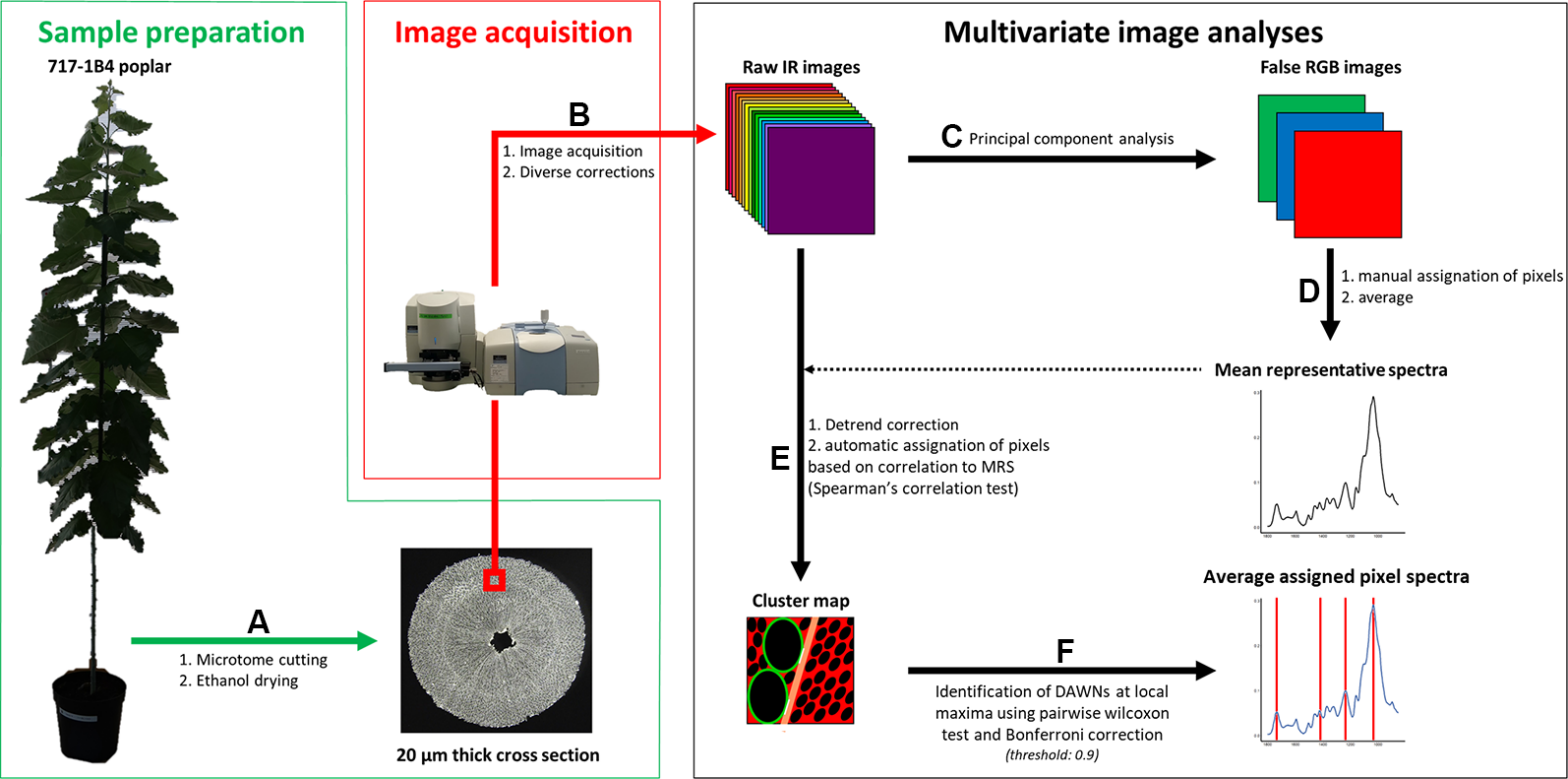
Workflow. Sample preparation. Poplar trees were grown in a greenhouse for three months. **(A)** 20 µm-thick ethanol dried cross section were produced. Image acquisition. **(B)** (100 x 100) µm² images were acquired from 20 µm thick cross sections using an infrared (IR) microscope. Background subtraction, noise reduction and atmospheric correction were applied on raw IR images using the SpectrumImage software (v.1.6.4). Multivariate image analyses were carried out using homemade scripts encoded in R (v. 3.4.4). **(C)** Principal component analyses (PCA) were performed on raw spectra. RGB intensities were derived from pixel contribution to PC1 for Red, PC2 for Green and PC3 for Blue. **(D)** Ninety pixels were manually attributed to each of ray, fiber and vessel cell walls and to G-layers. **(E)** Mean representative spectra (MRS), which is the pixel average of each of these classes, and pixel spectra were detrend. Correlations between pixel spectra and MRS were determined using Spearman’s correlation test. Pixel were assigned to a given class using a 0.9 correlation score threshold. Green: vessel, tan: ray, red: fiber, black: lumen, white: NA. **(F)** Differentially absorbed wavenumbers (DAWNs) were identified using pairwise Wilcoxon test. A Bonferroni adjusted P-value of 0.001 was used as cut-off criterion.

We analyzed ten classes of cell wall corresponding to the existing associations between cell wall layers (G- or S-layers), cell types (fiber, ray or vessel) and wood types (TW, OW or NW): TW fiber G-layer, TW fiber S-layer, OW fiber S-layer, NW fiber S-layer, TW ray S-layer, OW ray S-layer, NW ray S-layer, TW vessel S-layer, OW vessel S-layer and NW vessel S-layer. Multivariate image analysis has been performed independently for each type of wood using R (v. 3.4.4) (**Figure 1**). Principal component analysis (PCA) was applied on raw spectra using PCA function implemented in FactoMineR package (v. 1.40). False RGB images were then obtained using quilt.plot (fields, v.9.6). Red, blue and green intensities were based on pixel contribution to first, second and third principal component, respectively. Based on these false RGB images, specific mean representative spectrum (MRS) were built for each cell wall class, from the average of 90 selected spectra (that is 30 spectra manually selected in one of the three images of each biological replicate of each wood type). Both MRS and pixel spectra were corrected using the detrend function implemented in the prospectr package (v. 0.1.3). Correlations between each pixel spectrum and MRS of its type of wood were determined using the Spearman correlation test implemented in cor.test (stats, v.3.5.2). The correlation score was used to assign pixels to a cell wall class or to lumen. A pixel was considered as lumen when its correlation scores with all MRS were below the threshold value of 0.90. Pixels with very similar correlation scores with all MRS (less than 1% difference) were classified as “not assigned” (NA). Finally, the pixel was assigned to the cell wall class whose MRS has the highest correlation score with the pixel.

### Identification of Differentially Absorbed Wavenumbers (DAWNs)

DAWNs were determined using the pairwise Wilcoxon test from the R stats package (v.3.5.2). A Bonferroni adjusted *P*-value of 0.001 was used as cut-off criterion. Only DAWNs located at a local maximum (± 3 cm^–1^) were considered for further analysis. Local maxima were identified on the average spectra resulting from all pixels assigned to a class of cell wall. A single wavenumber (σ) was considered as a local maximum when its absorbance was higher compared to σ-2 and σ+2 cm^–1^. The assignation of DAWNs to cell wall components was based on an *in-lab* database (**Table S1**) compiling data from the literature and from the Histochem database (Durand et al., 2019; https://pfl.grignon.inra.fr/shistochem/).

### Statistical Analysis

For each cell type, a PCA was carried out on the whole spectra to discriminate between the different types of wood. Loadings were calculated as follow: $Loadings=Eigenvector\cdot \sqrt{Eigenvalues}$. Due to the huge size of the data set, pixels corresponding to the cell wall of each cell type in each image were averaged to produce heatmaps using heatmap.2 function (gplots package, v. 3.0.1.1), scaling on columns. Heatmaps dendrograms were calculated considering Pearson’s correlation between wavenumbers as a distance.

## Results

### From Raw Infra-Red Images to Pixel Clusters of the Different Cell Wall Types

One of the most important steps of the workflow was to produce a good assignation of the pixels to the different cell types from the different types of wood. Classic clustering methods, such as hierarchical clustering based on principal components, Ascending Hierarchical Classification, k-nearest neighbor and k-means were not conclusive. The most effective clustering method was the assignation based on Spearman’s correlation score between pixel spectra and a mean representative spectrum (MRS) for each class of cell wall.

**Figure 2:**
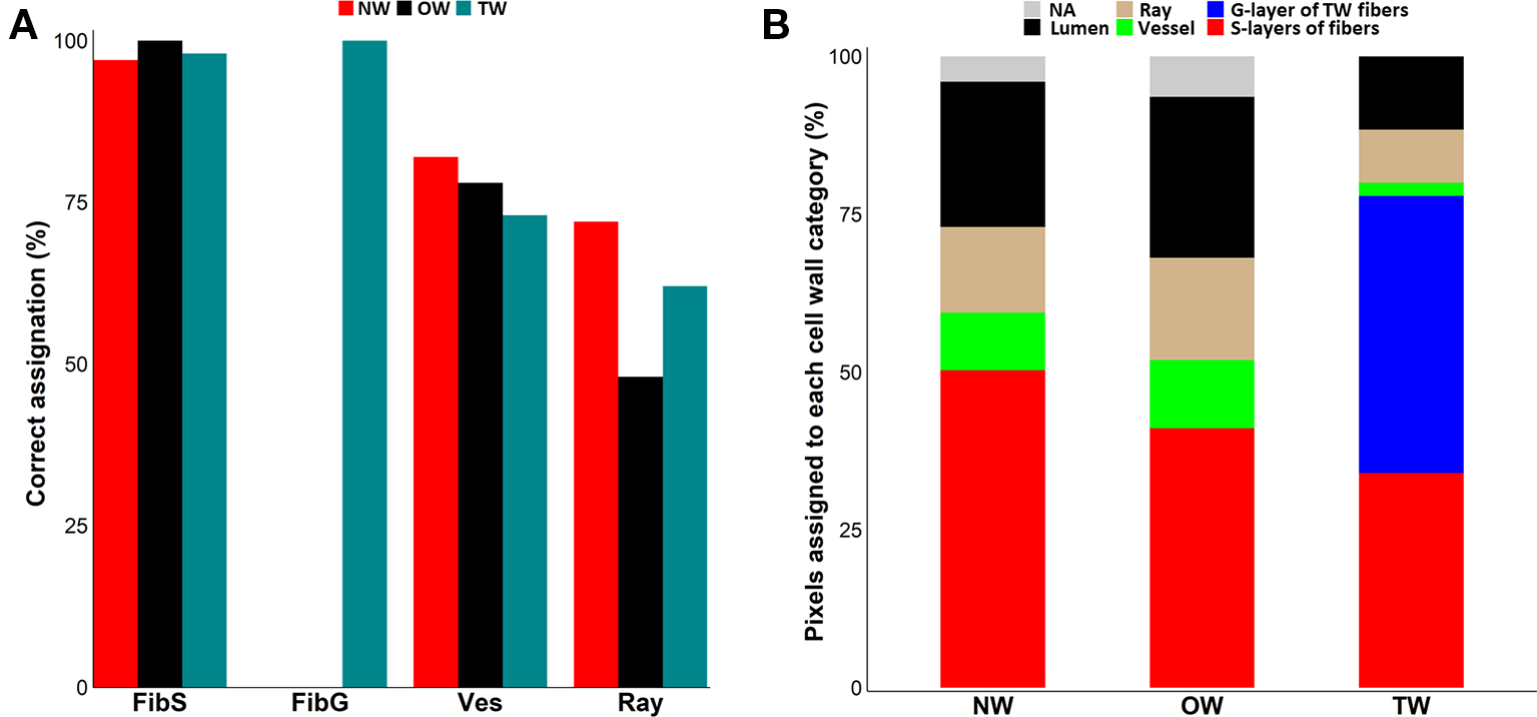
Assignation of pixel to cell-wall categories. **(A)** Clustering efficiency evaluated on 90 manually assigned pixels per cell wall type. Red: Normal wood (NW), Black: Opposite wood (OW), Pigeon blue: Tension wood (TW), FibS: fiber S-layers, FibG: TW fiber G-layers, Ves: vessel S-layers, Ray: ray S-layers. **(B)** Distribution of the 36,864 pixels in cell wall classes after assignation using Spearman’s correlation score between pixel spectra and mean representative spectra. NW, normal wood; OW, opposite wood; TW, tension wood; Red, fiber S-layers; Blue, TW fiber G-layer; Green, vessel S-layers; Tan, ray S-layers; Black, lumen; Grey, not assigned.

We applied this method to the spectra used to calculate the MRS of the different classes of cell wall (**Table S2**). When applied to pixels with a known assignment, our method correctly assigned 48% to 100% of the pixels depending on the cell wall category (**Figure 2A**). All the spectra from TW fiber G-layer were properly assigned, whereas, no spectrum from any TW S-layer class was wrongly assigned to the G-layer class. Likewise, all spectra of OW fiber S-layers were correctly assigned, while only three of them were identified as ray S-layers in NW and two of them were also identified as ray S-layers in TW. Our method correctly assigned a large part of vessel spectra: 78% in OW, 82% in NW and 73% in TW. In TW, nearly all wrongly assigned vessel spectra (23 out of 24) were classified as ray S-layers. In NW, only six vessel spectra were classified as ray S-layers, while four others had a correlation score below 0.90 with all MRS and were therefore classified as lumen. Finally, six spectra that presented similar correlation values to all MRS were in consequence not assigned. In OW, six vessel spectra were categorized as ray S-layers, three as fiber S-layers, four as lumen and seven were not assigned. Conversely, the wrong assignment of ray spectra was rather high reaching 52% in OW, 28% in NW and 38% in TW.

**Figure 3:**
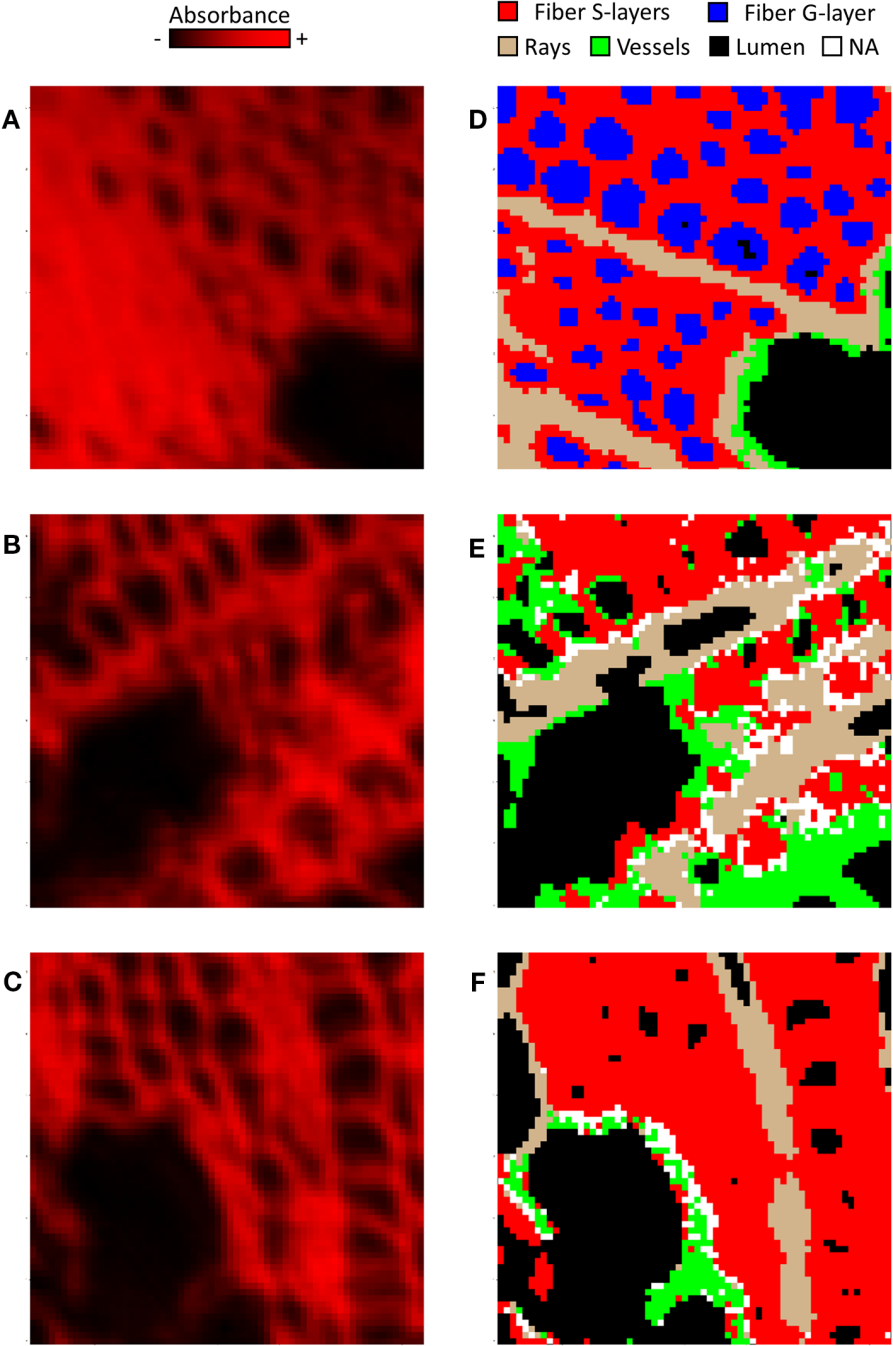
Comparison of raw images **(A–C)** and cluster map **(D–F)** of tension wood **(A, D)**, opposite wood **(B, E)** and normal wood **(C, F)** using one representative image per type of wood. Note that these images were not the ones used to determine MRS. **(A–C)** In raw images, the intensity in red reflects the mean absorbance of the pixel. **(D–F)** Red, fiber S-layers; Blue, fiber G-layer; Green, vessel S-layers; Tan, ray S-layers; White, NA; Black, lumen.

Subsequently, we used this workflow to assign the 36,864 spectra from the pixels of each type of wood. In NW, 50% of the spectra were categorized as fiber S-layers, 14% as ray S-layers, 9% as vessel S-layers and 23% as lumen, while 4% of the spectra could not be assigned. In OW, 41% of the spectra corresponded to fiber S-layers, 16% to ray S-layers and 11% to vessel S-layers, while 26% of the spectra, with a correlation score below 0.90, were considered as lumen. Finally, 6% of the spectra with similar correlation levels to MRS from all classes, were not assigned and therefore excluded from the analysis. In TW, the fiber G-layer was the largest class with 44% of the spectra. The fiber S-layers, ray S-layers, vessel S-layers and lumen categories were composed of 34%, 8%, 2% and 12% of the spectra, respectively. Interestingly, unlike OW and NW, all TW spectra have been assigned (**Figure 2B**). The comparison between the raw IR images and the corresponding cluster maps (**Figure 3**) revealed a correct adjustment especially for fibers and vessels, indicative of the efficiency of the clustering method used to predict these two cell types. The pixels that could not be assigned mainly corresponded to pixels at the interface between cell types. As the assignation for ray S-layers was not very efficient, IR profiling was restricted to fiber and vessel SCW.

### IR Profiling of Fiber and Vessel SCW

#### Fibers

**Figure 4:**
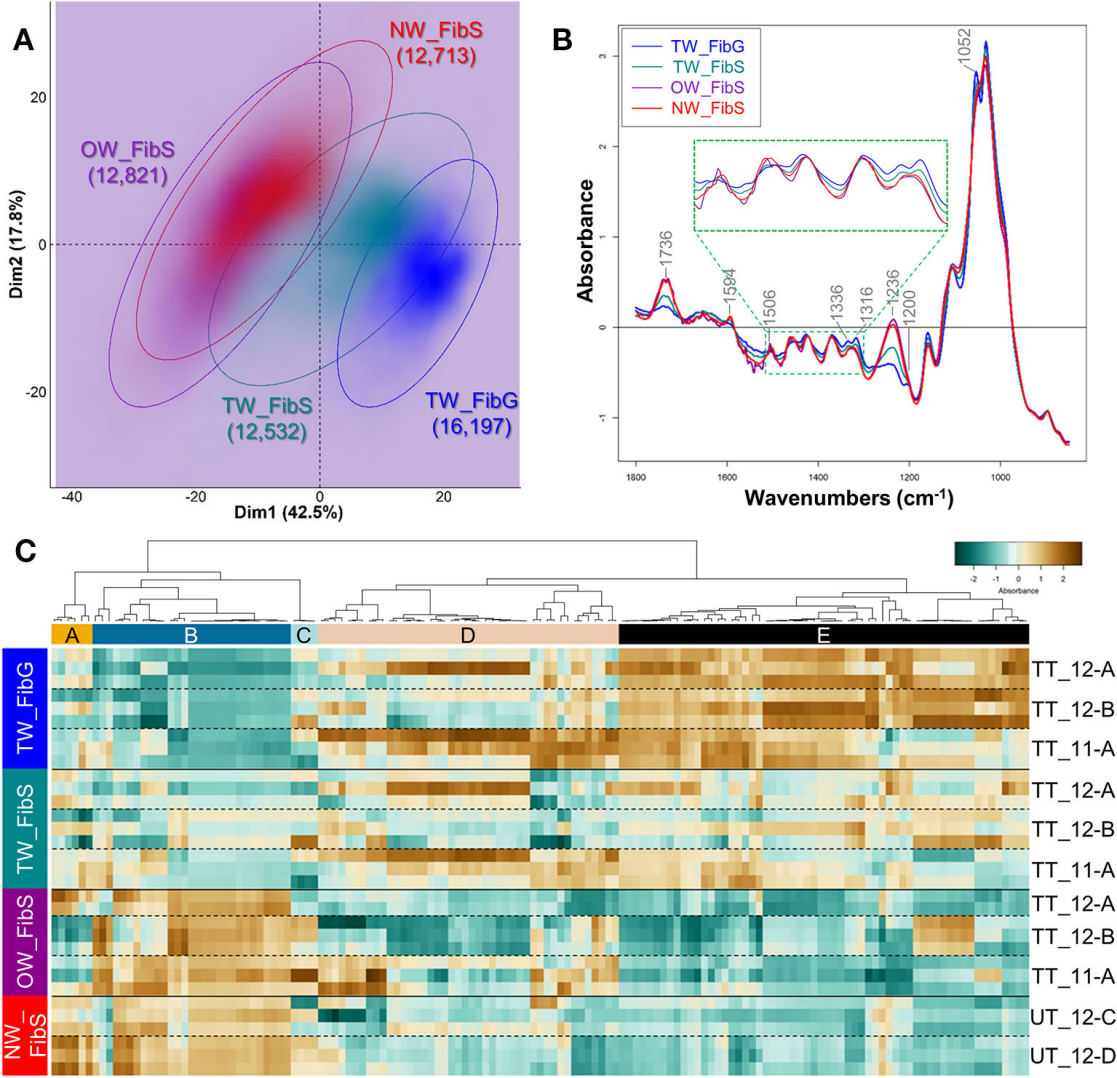
Chemical differences between the G-layer from tension wood fibers and the S-layers from fibers of tension, opposite and normal wood. **(A)** PCA score plot. Blue, TW fiber G-layer; Turquoise, TW fiber S-layers; Purple, OW fiber S-layers; Red, NW fiber S-layers. Ellipses encompass 95% of the data in a normal distribution. Numbers in brackets refer to the number of pixels assigned to the different cell wall categories. Color intensity reflects the density of individuals. **(B)** Average ATR-FTIR spectra. Blue, TW fiber G-layer; Turquoise, TW fiber S-layers; Purple, OW fiber S-layers; Red, NW fiber S-layers. The green dotted line insertion represents a zoom of the [1,510–1,300] cm^–1^ region. **(C)** Heatmap of differentially absorbed wavenumbers. TW_FibG, TW fiber G-layer, TW_FibS; TW fiber S-layers; OW_FibS, OW fiber S-layers; NW_FibS, NW fiber S-layers.

The fiber spectra, assigned by automatic clustering, were first analysed by PCA. When performed on all the images, PCA clearly discriminated three groups of spectra: TW fiber G-layer, TW fiber S-layers and a group combining spectra from both NW and OW fiber S-layers. However, in the latter group, two populations of spectra were detected, with the minor one gathering spectra all coming from the same 4 images (3 from NW and 1 from OW) (**Supplementary Figure S2**). These 4 images were therefore removed from the subsequent analysis as they may induce unwanted variability for technical or biological reasons. A new PCA was performed (**Figure 4A**), whose first dimension explained 42.5% of the variance and discriminated rather well TW fiber G-layer from the fiber S-layers of both OW and NW, whereas TW fiber S-layers stood in between. **Figure 4B** shows the average infrared spectrum of pixels assigned to the four types of fiber layers. The TW fiber G-layer spectrum shows significant changes compared to the OW and NW fiber S-layers spectra, including the disappearance of two bands at 1,594 and 1,506 cm^–1^, corresponding to aromatic skeletal vibration of lignins (**Table S1**) and the appearance of a well-resolved double band at 1,336 and 1,316 cm^–1^, that can be attributed to cellulose (**Table S1**, O-H in-plane bending and CH2 rocking vibration, respectively). It is also characterized by the appearance of a band at 1200 cm^–1^, that can be attributed to OH in-plane deformation in cellulose. This band is masked in the other S-layers because of the overlap of a more intense band at 1236 cm^–1^, corresponding to acetyl and carboxyl vibration in xylan and to C-C, C-O and C=O stretch in lignins. Finally, a last band is clearly visible in TW fiber G and S-layers at 1,052 cm^–1^, corresponding to C-O valence vibration, mainly from C3-O3H, most probably from cellulose. This latter band is detectable only as a shoulder on the spectra of NW and OW fiber S-layers. The spectrum of TW fiber S-layers is intermediate, in particular with weak bands at 1,506 cm^–1^, resembling the spectra of NW and OW; it also presents the G-layer characteristic bands at 1,136, 1,316, and 1,052 cm^–1^. In terms of absorbance differences, the bands at 1,736 and 1,236 cm^–1^ mainly differentiate TW and OW/NW fibers, with a higher absorbance in the latter (**Figure 4B**): they can be mainly attributed to acetylated xylans, and to a lesser extent to lignins (**Table S1**). The PCA loading plots underline the spectral features associated with the first and second dimensions, which are responsible for most of the variance (**Supplementary Figure S3A**). The samples were discriminated only by the first dimension (**Figure 4A**). The loading for PC1 is quite complex and cannot be easily interpreted by simple changes in few compounds. The loading is positive mostly for six ranges of absorbance bands (1,576–14, 1,492–74, 1,446–28, 1,412–1,384, 1,360–1,280, 1,014–992 cm^–1^). The first four ranges of bands do not correspond to known IR bands, while the two last groups may correspond to cellulose (crystalline cellulose at 1,335 cm^–1^ and 1,318–12 cm^–1^, C–O valence vibration at 996–85 cm^–1^) and lignins (1,330–24 cm^–1^, S ring plus G ring condensed). The loading is negative for four ranges of bands (1,758–08, 1,268–06, 1,136–16, 1,098–68 cm^–1^). The first three groups contain bands attributed to hemicelluloses, cellulose and lignins (**Table S1**). In order to identify more precisely differences between cell wall, we have identified DAWNs in the vicinity of a local maximum (± 3 cm^–1^), that were further used to generate a heatmap (**Figure 4C**, **Table S3**). The analysis was limited to these DAWNs because the most relevant biological information in an IR spectrum is carried by the wavenumbers at the level of the most intense bands. The heatmap shows 2 clusters of DAWNs that have a very contrasted absorbance profile between TW G-layer and S-layers of OW or NW fibers, while TW fiber S-layer has an intermediate profile. Cluster B gathers DAWNs with a high absorbance in NW and OW S-layers and a low absorbance in TW S- and G-layers. On the reverse, Cluster E groups DAWNs with a high absorbance in TW G-layer and to a lesser extent in TW S-layers and a low absorbance in NW and OW S-layers. Cluster B contains DAWNs in the range of 1,742–28 and 1,238–32 cm^–1^ related mainly to acetylated xylans, and in the range of 1,596–90 and 1,508–04 cm^–1^ related to lignins. Surprisingly, this latter group was weakly absorbed in NW fiber S-layers, compared to OW fiber S-layers. Cluster B also contains DAWNs in the range of 1,248–1,244 cm^–1^, that may be attributed to lignins (stretching of phenolics), and in the range of 1,464–58 cm^–1^, where both cellulose, lignins and xylans may contribute (CH2 of pyran ring symmetric scissoring; OH- and CH- deformation). Cluster E contains DAWNs that may correspond to crystalline cellulose (1,428–26, 1,336–1,314 cm^–1^, which appear as a double band in the TW G-layer spectra). Likewise, it contains DAWNs in the range of 1,282–78 and 1,162–56 cm^–1^ that may be also attributed to cellulose. Cluster E contains DAWNs in the range of 1,056–50 and 1,036–30 cm^–1^, that can be attributed to cellulose, hemicelluloses (and also to lignins for the latter group). Interestingly, it contains a group of DAWNs at 1,518–12 cm^–1^ that can be attributed to lignins (aromatic skeletal vibration, higher absorbance in G lignin in comparison to S lignin, **Table S1**) and one in the range of 1,556–48 cm^–1^ than can be attributed to proteins (Amide II: N-H deformation + stretching contribution from C-N stretching, **Table S1**). Finally, some groups of DAWNs cannot be easily related to known IR assignment (1,790–74, 1,696–92, 1,562, 1,540–30, 1,478–74 cm^–1^).

**Figure 5:**
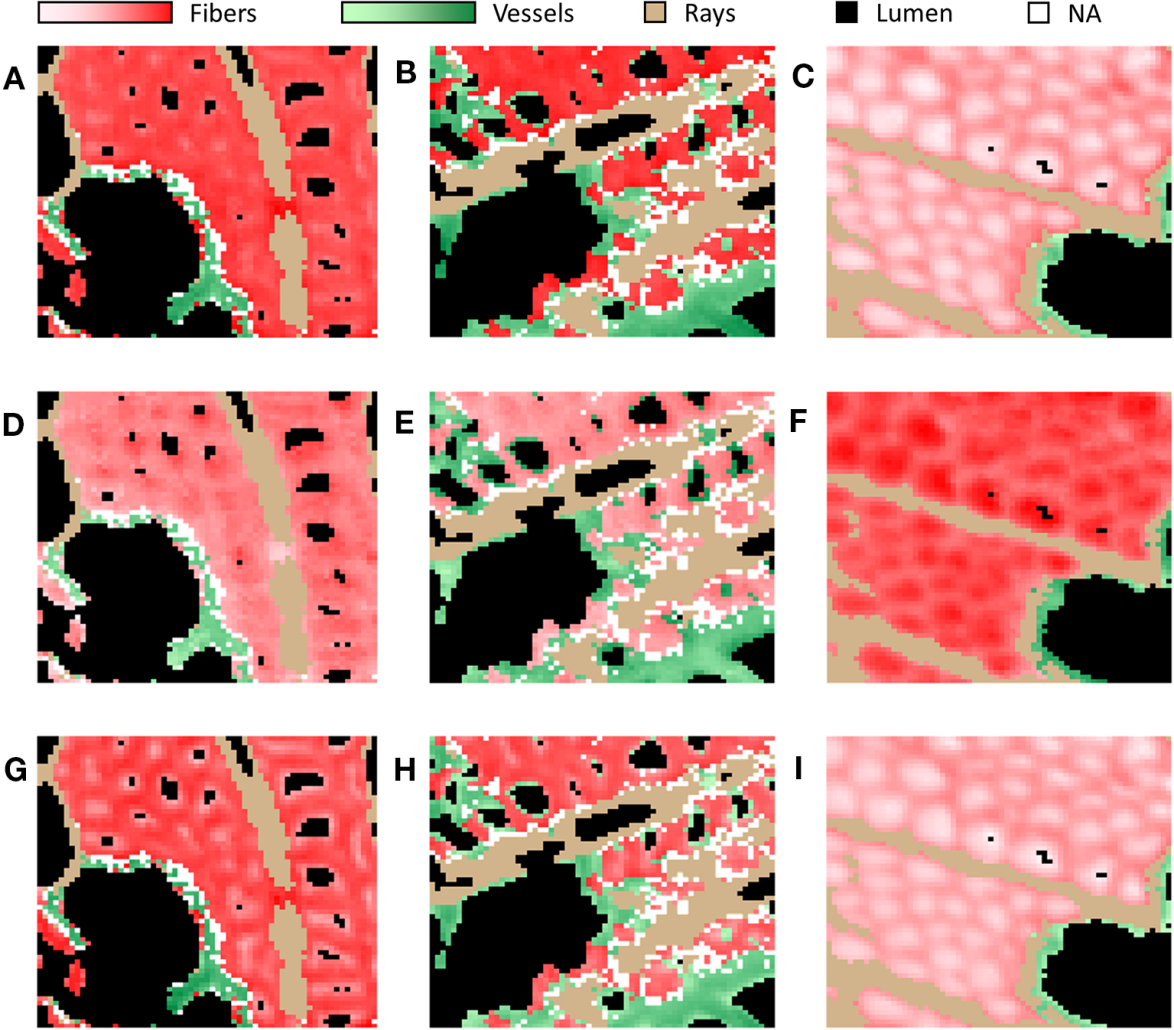
Infrared images of selected DAWNs of representative images of NW **(A, D, G)**, OW **(B, E, H)** and TW **(C, F, I)**. Selected DAWNs: 1,236 cm^–1^ corresponding to acetylated xylans and lignins **(A–C)**, 1,316 cm^–1^ corresponding to crystalline cellulose **(D–F)** and 1,736 cm^–1^ corresponding to acetylated xylans **(G–I)**. Red, fiber S-/G-layers; Green, vessel S-layers; Tan, ray S-layers; White, NA; Black, lumen. Red, and green intensities are proportionate to the absorbance of pixels assigned to fiber S/G-layers and vessel S-layers, respectively.

**Figure 5** shows images reconstructed on the basis of the absorbance level for each pixel at a given wavenumber. Three wavenumbers were chosen: 1,736 cm^–1^ (acetylated xylans), 1,316 cm^–1^ (crystalline cellulose), and 1,236 cm^–1^ (acetylated xylans and lignins). In particular, the images clearly show the absorbance gradient between fiber G-layer and S-layer in TW.

#### Vessels

**Figure 6:**
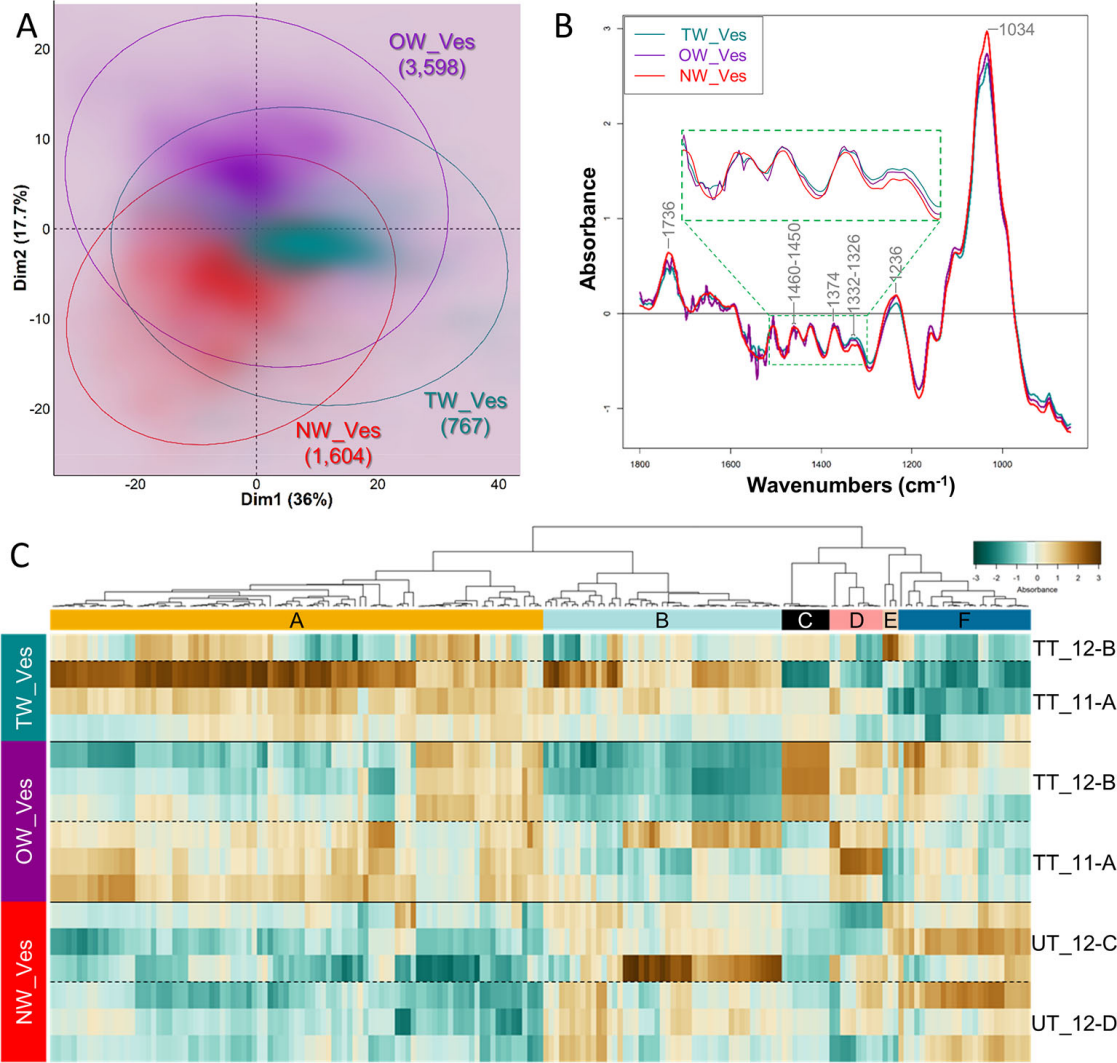
Chemical differences between vessel S-layers of tension, opposite and normal wood. **(A)** PCA score plot. Turquoise, TW vessel S-layers; Purple, OW vessel S-layers; Red, NW vessel S-layers. Ellipses encompass 95% of the data in a normal distribution. Numbers in brackets refer to the number of pixels assigned to the different cell wall categories. Color intensity reflects the density of individuals. **(B)** Average ATR-FTIR spectra. Turquoise, TW vessel S-layers; Purple, OW vessel S-layers; Red, NW vessel S-layers. The green dotted line insertion represents a zoom of the [1,500–1,300] cm^–1^ region. **(C)** Heatmap of differentially absorbed wavenumbers. TW_Ves, TW vessel S-layers; OW_Ves, OW vessel S-layers; NW_Ves, NW vessel S-layers.

PCA analysis on vessel S-layers spectra slightly discriminates the spectra from TW, OW and NW, according to the first two dimensions, explaining 36 and 17.7% of the variance (**Figure 6A**). When the first dimension partially separates TW vessel S-layers from both OW and NW vessel S-layers, the second one slightly distinguishes OW and NW vessel S-layers. The average infrared spectra of the vessel S-layers from the three types of wood appear rather similar (**Figure 6B**). The average spectra of OW and TW vessel S-layers exhibit an important noise between approximately 1,800 and 1,450 cm^–1^. Nevertheless, we can clearly distinguish in these two spectra, three double bands at 1,736, 1,460, and 1,374 cm^–1^, while there is only one band in the NW spectrum at these wavenumbers. Acetylated xylans mainly contribute to the first band and cellulose, hemicelluloses and lignins to the two others. The bands at 1,736 (acetylated xylans), 1,332–26 (lignins, S ring plus G ring condensed), 1,236 (acetylated xylans and lignins) and 1,034 cm^–1^ (cellulose and lignins) makes possible to differentiate TW, NW and OW vessel S-layers (**Figure 6B**). The PCA loading plots (**Supplementary Figure S3B**) show that on PCA first dimension, the loading is positive for four spectral zones (1,580–26, 1,494–74, 1,450–1,380, and 976–904 cm^–1^), that cannot be easily assigned to reported IR bands except the region of 1,430–21 cm^–1^ that can be assigned to cellulose, hemicelluloses and lignins (**Table S1**), 996–85 cm^–1^ to cellulose and 925–15 cm^–1^ to lignins. The loading is negative for three ranges of bands (1,760–22 cm^–1^, 1,256–36 cm^–1^ and 1,138–1,034 cm^–1^), corresponding respectively to lignins and acetylated xylans, lignins, cellulose and lignins (**Table S1**). On the second dimension, two main ranges of bands (1,800–1,756 cm^–1^ and 1,206–1,164 cm^–1^) presented positive loadings, the second one being potentially attributed to cellulose, hemicelluloses and lignins (**Table S1**). The loading is negative mainly for two groups of bands (1,676–56 and 1,650–1,590 cm^–1^). They both correspond to lignins (C=O stretch in conjugated p-substituted aryl ketones/aromatic skeletal vibrations, higher absorbance in G lignin in comparison to S lignin, **Table S1**). As done for fiber analysis, the DAWNs in the vicinity of a local maximum (± 3 cm^–1^) were used to generate a heatmap (**Figure 6C**, **Table S4**). The heatmap shows more variability between samples, compared to the heatmap for fiber, most likely because vessel/fiber area ratio is small in wood and in consequence, there were fewer vessel pixels available. Cluster A corresponds to DAWNs that have a low absorbance in NW vessel S-layers, and a high absorbance in TW vessel S-layers, while the absorbance is generally contrasted between the two sections of OW analyzed. Cluster A contains DAWNs in the range of 1,364–60 and 1,346–42 cm^–1^, that may be mostly attributed to cellulose and in the range of 1,336–22 cm^–1^, that may be attributed to crystalline cellulose and also to lignins (S ring plus G ring condensed). DAWNs of 1,374–72 cm^–1^ may be attributed both to cellulose and hemicelluloses (CH deformation vibration and CH bending, **Table S1**). The isolated DAWN of 1,506 cm^–1^ can be assigned to lignins (aromatic skeletal vibrations; G > S). Besides, a large number of DAWNs cannot be easily related to known IR assignment (1,686–82, 1,572–68, 1,560–58, 1,538–28, 918, 914–10, 936–26 cm^–1^). Cluster F gathers DAWNs that are less absorbed in TW vessel S-layers, when compared to OW and NW vessel S-layers. DAWNs in the range of 1,236–32 cm^–1^ correspond to acetylated xylans. The 3 groups of DAWNs in the same region of the IR spectra (1,744–36, 1,732–26, 1,722–10 cm^–1^) are due to C=O stretching, that are mainly due to acetylated xylans too, but the involvement of esterified lignins cannot be excluded (**Table S1**). On **Figure 6B**, the spectrum for NW shows a unique band around 1,734, while several bands and shoulders can be seen in TW and OW, probably related to a decreased amount of xylans, making visible other individual bands. Finally, both cellulose and lignins may contribute to DAWNs in the range of 1,040–32 cm^–1^ (C–O valence vibration, mainly from C3–O3H; aromatic C-H in-plane deformation, G > S).

## Discussion

In this study, we set up a non-destructive method for the microphenotyping of wood cell walls, based on ATR-FTIR microspectroscopy, that makes possible to obtain, in a rather short time, IR spectra from the different wood cell types, thus overcoming the complexity of wood tissue. We have developed a high-throughput pipeline for hyperspectral image analysis (**Figure 1**) with both automatic pixel clustering according to cell wall types and identification of DAWNs between samples. This pipeline was used to compare the biochemical composition of the cell wall from both fibers and vessels originating from three kinds of wood in poplar: normal wood of upright trees, tension and opposite wood of artificially tilted trees. As the preparation of the samples is very simple—microtome sectioning at 20 µm, followed by ethanol drying—the acquisition of the images is fast—40 min for a (100 x 100) µm^2^ hyperspectral image—and the analysis is mostly an automated process, this method is suitable to screen for differences in IR spectra, a large number of samples, at the fiber or vessel cell walls. Thanks to the high refractive index of the Germanium crystal (4.01), the spatial resolution of ATR-FTIR microspectrocopy is 3.1 µm, and based on an oversampling factor of two, it is possible to reach a pixel resolution of (1.56 x 1.56) µm^2^. This is sufficient to get IR spectra at the level of secondary cell walls, and with respects to the possibility of signal contamination, we obtained the best results for fiber cell walls, due to their higher thickness compared to vessel and ray cell walls. However, this method is not resolutive enough to reach the level of the cell wall layer. For such studies, some other methods of IR nanospectroscopy, such as scattering scanning near-field optical microscopy (s-SNOM) or atomic force microscopy infrared nanospectroscopy (AFM-IR), would be more appropriate. Indeed, the resolution of such methods is beyond the classical diffraction limit of light. These techniques were recently used to investigate the chemical composition of primary cell walls in *Populus* vascular cambium (Pereira et al., 2018). The drawback of these techniques is that ultrathin sections need to be prepared, with potential chemical treatments like DMSO that may alter cell wall composition, and therefore IR spectra.

A critical step to identify differences in cell wall composition using ATR-FTIR microspectroscopy is to unequivocally assign pixels to the right type of cell wall. The clustering method used here makes possible such an assignment with a high efficiency for both fiber S- and G-layers and with an acceptable level of success for vessels. The low score for the assignation of ray pixels probably results from the high diversity in ray cell walls. Indeed, as rays are uniseriate in *Populus* (IAWA Committee, 1989), there are very few ray-to-ray cell walls on transverse sections and most ray cell walls are either neighbored by fiber or vessel cell walls. Therefore, in this study, the pixels classified as ray cell walls correspond in fact to a mixture of ray/vessel and ray/fiber cell walls, whose composition may differ accordingly. Ray cells can be classified into three different cell types, named contact cells, intermediate cells and isolated cells (Braun, 1967; Nakaba et al., 2012) that may have cell walls with rather different features. While contact cells are located predominantly within the upper and lower lines of individual ray lines and are connected to adjacent vessel elements through pits, intermediate cells are located within the same radial lines but are not adjacent to vessel elements. Isolated cells are located within the radial cell lines with no connections with vessels (Braun, 1967). In *Populus sieboldii x P. grandidentata*, it has been shown that secondary cell wall thickening is initiated earlier in the upper and lower cell lines of a ray (Nakaba et al., 2012). In addition, parenchyma ray cells stay alive longer than vessels and fibers and their cell wall composition may also be subjected to modifications according to their age. These aspects may be at least partly circumvented by applying ATR-FTIR technique on longitudinal sections in place of transverse sections. The score for the assignation of vessel pixels is not as high as for fiber pixels: as for rays, vessel pixels may in fact correspond to a mixture of vessel/vessel, vessel/ray and vessel/fiber cell walls. The fact that vessels have thin cell walls may also contribute to some incorrect assignments. Finally, the lower score for the assignment of pixels in OW compared to NW and TW (**Figure 2B**) may be linked to the reduced growth of xylem cells resulting from the position of OW on the lower side of the tilted stem. The smaller number of pixels assigned to vessel cell wall in TW compared to OW and NW is in accordance with the observations from a number of studies using other approaches, that the number of vessels is decreased in TW in comparison to OW (Jourez et al., 2001; Ruelle et al., 2006; Tarmian et al., 2009) or to NW (Chow, 1947). We also observed in TW a decreased number of lumen-associated pixels, which certainly result in the reduced lumen size due to the presence of the G-layer (Clair et al., 2006; Ruelle et al., 2006; Déjardin et al., 2010; Gorshkova et al., 2010). Finally, the smaller number of pixels assigned to fiber S-layers in TW supports the fact that S-layers are reduced in G fibers, in relation to the deposition of the G-layer.

ATR-FTIR microscopy provided IR profiling of fibers and vessel cell wall layers and DAWNs were identified and used to characterize differences in biochemical composition between layers. Another critical step was to correctly assign these DAWNs to wood polymers as the absorption band from a given functional group may originate from several wood polymers and bands are overlapping in the spectrum. This is the reason why we focused on the DAWNs in the vicinity of visible peaks on the spectra to get information easier to interpret on a biological level. TW and G fibers have been extensively studied using various complementary methods (FTIR, Raman imaging, biochemical analyses, immunolocalization). These are valuable data to validate our results using *in situ* IR profiling. In this study, the IR spectra of TW fiber G-layer is indeed very similar to what was obtained by Olsson et al. (2011) on TW and isolated G-layers, with a large decrease of the bands at 1,736 and 1,236 cm^–1^, no bands at 1,594 and 1,506 cm^–1^, and the appearance of 3 specific bands at 1,136, 1,316, and 1,200 cm^–1^. Basically, it has also the same profile as the one presented in Gierlinger et al. (2008), except that we did not observe, the large band at 1,645 cm^–1^ attributed to the deformation of vibration of adsorbed and free water: this probably reflects a difference in the water status of the samples analyzed. Our results indicate that TW fiber G-layers contained higher amounts of crystalline cellulose and lower amounts of acetylated xylans and lignins than fiber S-layers, although it is not so striking for TW S-layers (see supra for discussion on this). This is consistent with a number of studies demonstrating that the G-layer was mainly composed of cellulose (e.g. Norberg and Meier, 1966; Nishikubo et al., 2007; Guedes et al., 2017). This is also in accordance with the absence or the low levels of xylans and lignins reported elsewhere. The higher absorbance of a DAWN attributed to proteins is relevant since arabinogalactan proteins were reported as very abundant in TW in a number of studies (e.g. Lafarguette et al., 2004; Andersson-Gunnerås et al., 2006). Interestingly, while most lignin specific DAWNs exhibited lower absorbance in the G-layer, one of them presented a higher absorbance (1,518–12 cm^–1^, aromatic skeletal vibration, higher absorbance in G lignin in comparison to S lignin). In addition, a positive loading on the first dimension of PCA was found for a group of bands corresponding also to lignins (1,330–24 cm^–1^, S ring plus G ring condensed). This suggests that some lignins, likely different from the fiber S-layer lignins, are actually present in the G-layer, as previously reported in poplar (Joseleau et al., 2004; Gierlinger and Schwanninger, 2006) and in other species (Ghislain et al., 2016; Higaki et al., 2017). Thus, the *in situ * technique described in this paper is validated on TW fiber G-layers, since the results we obtained on individual G-layers were in accordance with other studies made at the tissue level or on isolated G-layers.

Regarding fiber S-layers, many DAWNs exhibited important differences in absorbance between TW fiber S-layers and fiber S-layers from OW or NW. From our observations, the former seemed to have a biochemical composition rather similar to what is described in the G-layer, while some features remained common to the S-layer from OW and NW. For example, the absorption bands for lignins and acetylated xylans were present in TW fiber S-layers but lower than in OW and NW. These observations may reflect biological differences in fiber cell wall assembly between the different wood types. This may be related to the fact that the S-layers in G fibers are not fully developed, in comparison to the S-layers of OW and NW fibers. Alternatively, it remains possible that the induction of tension wood formation, beside inducing G-layer formation, may also act on S-layer differentiation. However, we cannot rule out that these differences result from signal contaminations by the adjacent G-layer. Therefore, it remains to verify with a larger number of observations that our results reflect the biological reality or if the close proximity of the G-layer interferes with the measurements.

IR profiling was also obtained for vessel S-layers. The two first dimensions of PCA were able to discriminate between the three types of wood (**Figure 6A**) showing that there are some chemical differences in the cell wall of vessels, even between NW and OW, while fiber S-layers were very similar for both types of wood. The analysis of DAWNs between the different kinds of wood was not easy. Indeed, there was an important variability between the different sections analyzed [see for example differences between TT_11A and TT_12B, the 2 replicates for OW vessel cell wall (**Figure 6C**)]. This certainly reflects the low number of pixels analyzed (in comparison to fibers) that mainly results from the fact that vessels have thin cell wall and are not numerous (still in comparison to fibers). As for fiber cell walls, there are obviously important differences between vessel cell wall from NW and TW, with more cellulose and less acetylated xylans in the latter, two features also observed in TW fiber cell wall. Although, G-layer is absent from vessel cell wall, we cannot rule out a response at the vessel cell wall to stem tilting. The differences between NW and OW vessel S-layers, based on PC2 loadings, are mainly explained by differences in lignins. As we did not find any information on vessel cell wall composition in the bibliography, it is difficult to go further on these observations without performing measurements on a larger number of samples or using complementary techniques like immunocytochemistry.

## Conclusion

Thanks to the high pixel resolution of ATR-FTIR hyperspectral images—(1.56 x 1.56) µm² —we were able to get IR profiling of fibers and vessels at the cell layer level. We have developed a high-throughput pipeline for hyperspectral image analysis with both automatic pixel clustering and identification of differentially absorbed wavenumbers between samples. We have implemented this pipeline on different types of wood, including tension wood, a model that has been extensively characterized from a biochemical point of view. We confirmed previously known results on G layers, which validates this *in situ* approach, and we generated an IR profile for vessel S-layers in 3 different types of wood, as well as for TW fiber S-layers, providing information on the composition of cell layers rarely described in the literature. This methodological study paves the way for the microphenotyping of cell walls to rapidly and finely characterize various genetic resources or trees submitted to various stresses, and thus to overcome the complexity of wood tissue for all studies aimed at understanding wood formation. We consider that this technique is useful to give some clues about the cell wall compounds underlying cell wall differences. However, with regards to the inherent limitations of this technique (spatial resolution, difficulties to unambiguously assign bands to specific cell wall compounds), the hypotheses raised have to be validated by a complementary low-throughput technique, like immunocytochemistry.

## Data Availability Statement

The raw data supporting the conclusions of this article will be made available by the authors, without undue reservation, to any qualified researcher.

## Author Contributions

CC, FL, GP, and AD designed the research. CC, PM, FL, CG-P, and VL-P acquired the data. CC, PM, and AD analyzed the data. CC, GP and AD wrote the article.

## Funding

This research was funded by Centre Val de Loire Region, APR-IR #2016-00108472 (OPeNSPeNU).

## Conflict of Interest

The authors declare that the research was conducted in the absence of any commercial or financial relationships that could be construed as a potential conflict of interest.

## Supplementary Material

Supplementary figure 1 Crystal impacts on UT_12-C cross-section. *Green circles pinpoint the three impacts*.

Supplementary figure 2 PCA score plot of fibres on the whole data set. *Blue: TW fibre G-layer, Turquoise: TW fibre S-layers, Purple: OW fibre S-layers, Red: NW fibre S-layers. Ellipses encompass 95% of the data in a normal distribution. Color intensity reflects the density of individuals*.

Supplementary figure 3 PCA loading plots of fibre S- and G-layers **(A)** and vessel S-layers **(B)**. *Red: PC1, Green: PC2*.
