# Supplementary figures and images for: ATR-FTIR Microspectroscopy Brings a Novel Insight Into the Study of Cell Wall Chemistry at the Cellular Level
Source: Front Plant Sci. 2020 Feb 21;11:105. doi: 10.3389/fpls.2020.00105 (PMC7047332; doi:10.3389/fpls.2020.00105)

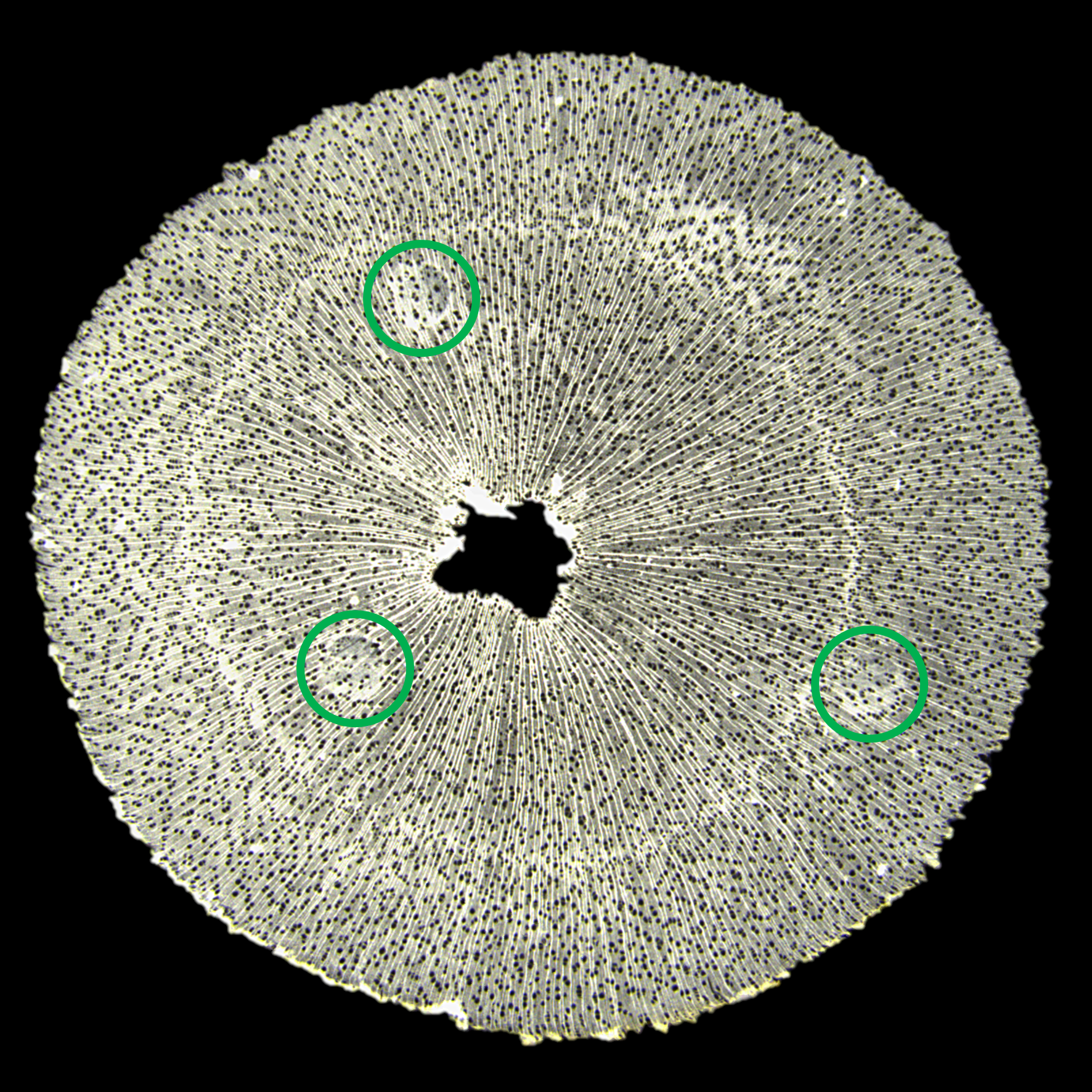

Supplement: Supplementary figure 1 — Crystal impacts on UT_12-C cross-section. Green circles pinpoint the three impacts. [file Image_1.tif]

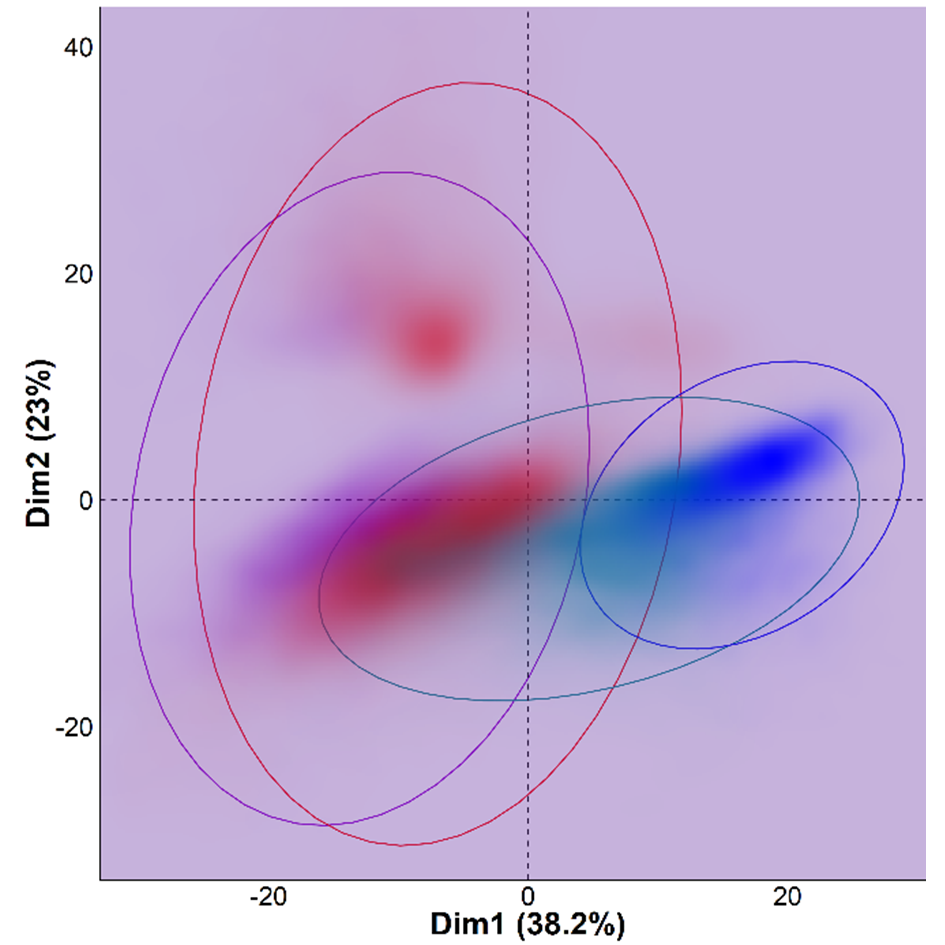

Supplement: Supplementary figure 2 — PCA score plot of fibres on the whole data set. Blue: TW fibre G-layer, Turquoise: TW fibre S-layers, Purple: OW fibre S-layers, Red: NW fibre S-layers. Ellipses encompass 95% of the data in a normal distribution. Color intensity reflects the density of individuals. [file Image_2.tif]

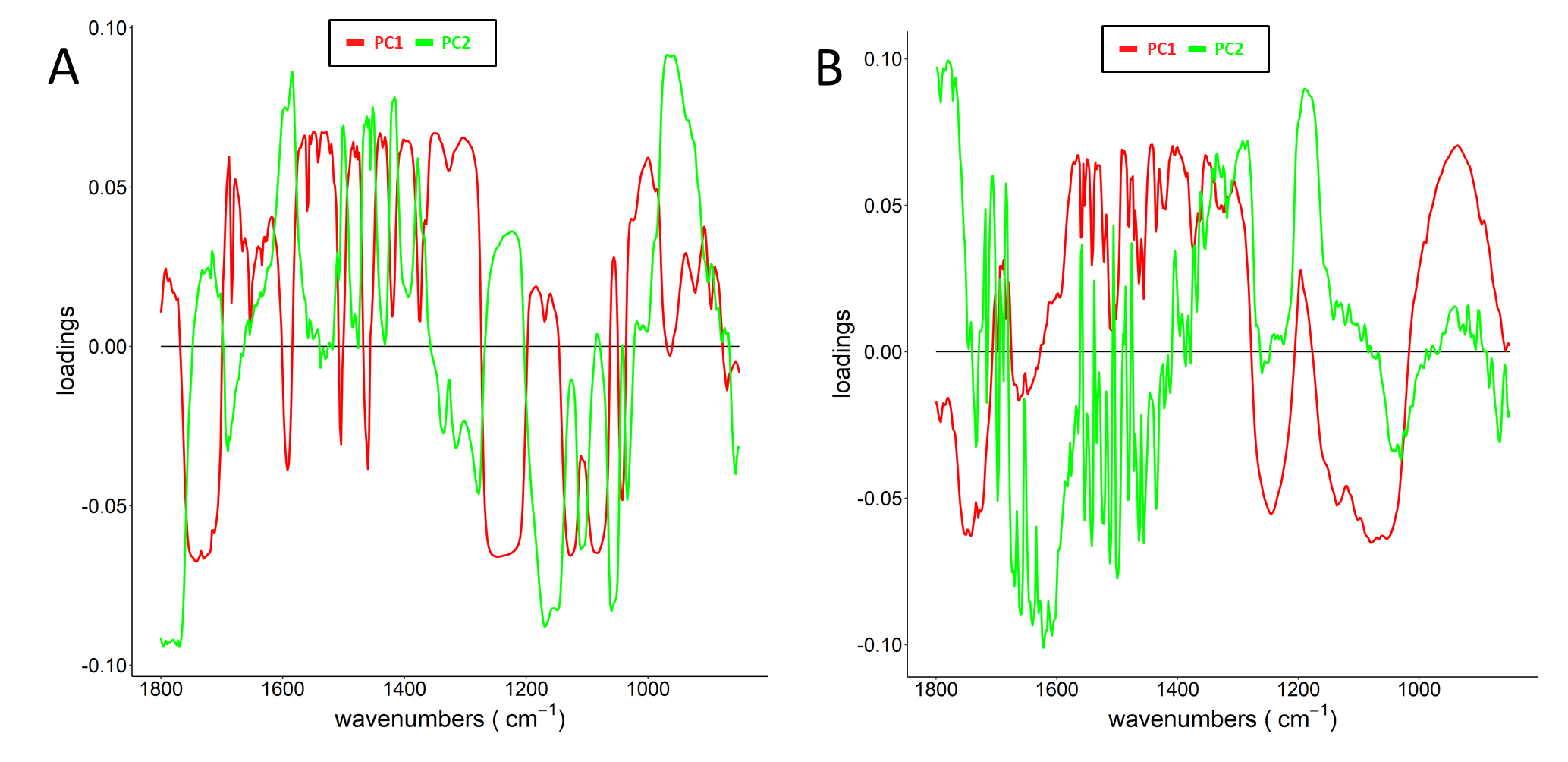

Supplement: Supplementary figure 3 — PCA loading plots of fibre S- and G-layers (A) and vessel S-layers (B). Red: PC1, Green: PC2. [file Image_3.tif]
